# Harvey Cushing and Sigmund Freud shaking hands: How electrical brain stimulation became a psychoanalytic method to study the unconscious (1870–1955)

**DOI:** 10.1177/0957154X251318494

**Published:** 2025-03-13

**Authors:** Max van der Linden, Richard Ridderinkhof

**Affiliations:** Department of Psychology, University of Amsterdam, The Netherlands; Faculty of Social and Behavioural Sciences, University of Amsterdam, The Netherlands; Department of Psychology, University of Amsterdam, The Netherlands; Faculty of Social and Behavioural Sciences, University of Amsterdam, The Netherlands

**Keywords:** Electrical brain stimulation, evolutionary theory, neurophysiology, neurosurgery, psychoanalysis

## Abstract

In the first half of the 1950s, psychoanalysts and neurosurgeons used electrical brain stimulation to explore hard-to-reach, unconscious psychological processes such as repressed memories, defence mechanisms and sexual identity. The development of evolutionary theory and neurophysiological methods and theory, together with the birth of psychoanalysis, were important precursors to these remarkable stimulation experiments. Experimental, theoretical and clinical antecedents of these stimulation experiments between the 1870s and the 1940s are discussed to show how smoothly the apparently opposing perspectives of psychoanalysis and neurophysiology merged. Two case studies are then briefly described. It is concluded that this striking and brief collaboration demonstrated a pragmatic and eclectic approach that integrated different theories and methods for a more holistic understanding and treatment of psychiatric disorders.

## Introduction: Brain research on psychoanalytic processes

One of the historical interpretations of the evolution of psychiatry is that of the pendulum swing (Richert and Reilly, 2014). Over time, according to this view, the centre of gravity of the orientation on cause, diagnosis and treatment within psychiatry has shifted several times. In the second half of the 19th century, for instance, a biological perspective was dominant within academic psychiatry. After the Second World War, psychoanalysis was highly influential in psychiatry, despite internal struggle for direction and growing (external) criticism of its scientific and clinical merits (Hammett, 1965; Tsikandilakis et al., 2019: 2; Wallerstein, 2002). From the 1980s, evidence-based medicine and the biological perspective regained dominance. According to some historians of psychiatry, this history even took the form of a struggle between two irreconcilable camps with a biological approach on one side and a social-psychological or psychoanalytic approach on the other (Shorter, 1997).

From the turn of this century, a growing group of historians of psychiatry began to agitate against an overly absolute metaphor of the pendulum swinging back and forth (Metzl, 2003; Pickersgill, 2010; Pressman, 1998; Rasmussen, 2006; Raz, 2008; Sadowsky, 2006). Many saw this metaphor as a historically useful heuristic with some realism; yet this metaphor also gave too one-sided a representation of psychiatry’s history. The biological and social-psychological and psychoanalytic approaches to psychiatric disorders do not behave like oil and water over time, but have always also mixed to a greater or lesser extent. Through the use of psychoanalytic insights in the application of (ablative) psychosurgery, electroconvulsive therapy, personality disorders and psychopharmaceuticals, they have looked ‘beyond the metaphor of the pendulum’ and shown that an important part of psychiatric practice has always been more diverse, more eclectic, pragmatic and less polarised (Sadowsky, 2006).

In the first half of the 1950s when psychoanalysis developed its dominance in the United States, a remarkable small-scale collaboration between some American neurosurgeons and psychoanalysts took place. The effects of electrical brain stimulation in some patients during neurosurgical surgery – from simple bodily reactions to specific memories and abstract symbolic experiences – awakened curiosity among some psychoanalysts. What caused these patients to have these kinds of experiences? What mental processes underpinned them? How were they related to brain processes? Again, therefore, the historical assumption of irreconcilability between the biological-psychiatric perspective and psychoanalysis does not appear to hold.

This article describes these stimulation experiments and examines the historical background of their development. From about 1870, a new understanding about the functioning of the brain began to emerge, in which a dynamic relationship between evolutionary older unconscious emotional processes and evolutionary newer conscious rational processes was central. These ideas formed the basis of more systematic empirical research on emotions, emotional behaviour and psychosomatics from the mid-1920s onwards. Doctors and researchers not much later also related psychoanalytic concepts to this vertical view of brain functioning, trying to explain the effects of biological treatments, such as electroconvulsive therapy (ECT). That laid an inspiring foundation for using direct electrical stimulation of the brain to do psychoanalytic research.

## Vertical Victorian brains

During the Victorian era (1837–1901), the UK enjoyed a stable and prosperous period. The middle class grew thanks to profits from colonies and domestic industrial developments. For this middle class, moral control was a central concept (Smith, 1992: 27–28). Man had to work hard to create a Victorian mind in order to maintain control over the forces of lust, disorder and insanity, which were ubiquitous in nature, through the will (Barlow, 1843).

These psychological ideas of control were then also applied to the functioning of the brain (Harrington, 1987; Stiles, 2006). The anatomical structure of this organ provided opportunities to explain the unruly tension between healthy and unhealthy forces. In 1844, for instance, Bristol-based general practitioner Arthur Wigan published a book with the lengthy title *A new view of insanity: The duality of the mind proved by the structure, functions, and diseases of the brain & by the phenomena of mental derangement, and shown to be essential to moral responsibility*, in which he used differences between the left and right hemispheres to explain the experience of diseased and healthy forces in the mind (Wigan, 1844). The brain was a ‘double’ brain with a ‘good’ and a ‘bad’ hemisphere. Wigan placed great emphasis on training and cultivating the brain to develop more self-control and moral responsibility (Ortega, 2011).

Hemispheric explanations were – viewed from the organisation of the brain – horizontal explanations. Influenced by the evolutionary theory of Charles Darwin (1809–1882), vertical brain models emerged in the last quarter of the 19th century to explain (neurological) disease, insanity or the always brewing low drives. The British neurologist John Hughlings Jackson (1835–1911) developed an influential theory based on the premise that the central nervous system had sensorimotor units at different levels (Hughlings Jackson, 1873). These units processed sensory information and converted it into behaviour. The simplest units were at the bottom of the brain. The most complex ones were at the outermost regions of the brain, in the cortex. Throughout the evolutionary history of the brain, newer areas had developed on top of the older ones. These ‘higher’ areas integrated information from simple areas into more complex and flexible conscious units of information. Even several important psychological functions in the cortex, such as will, memory, reason and emotion, found their origin in sensorimotor units (Hughlings Jackson, 1888: 365).

According to Hughlings Jackson, the higher areas controlled the lower areas. This insight added value to the diagnosis of neurological disorders. A key idea of Hughlings Jackson was that damage to a brain area could not produce positive effects, but only negative ones. When a particular area of the brain was lost due to damage, the function of that area was also lost. Therefore, if novel behaviour was observed after damage in the cortex in a laboratory animal or patient, it had to come from lower brain areas. The damaged higher cortical brain areas could no longer adequately perform their controlling function and the lower primitive brain areas were given free rein. They were released from cortical control.

Another idea that Hughlings Jackson developed was the process of dissolution, the opposite of evolution. In many neurological disorders and (psycho)pathological processes, such as drunkenness, the higher regions are affected first. The person then descended into a lower form of thought and action. The idea of dissolution was also relevant to psychiatry, according to Hughlings Jackson (Hughlings Jackson, 1889: 496). To him, various psychiatric disorders were diseases of the highest cerebral centres and were caused by various forms of dissolution.

The interaction between activity of the brain, of other organs, other bodily processes and emotions received increasing scientific attention in the 1880s. American philosopher William James (1842–1910) had concluded in 1884 that conscious emotions arose by reading off bodily changes (James, 1884). According to James, each emotion had its own constellation of bodily changes (such as blood pressure, pupil dilation, digestive organ activity and muscle tension) in response to an emotional stimulus from the environment. Only the perception of those bodily sensations by the cortex provided the experience of the emotion. Without the bodily changes, according to James, the perception was ‘pale, colourless, destitute of emotional warmth’ (James, 1884: 190). An emotion did not even exist without bodily sensations: ‘a purely disembodied human emotion is a nonentity’ (James, 1884: 194). It was mainly the activity of the intestines (viscera) that provided emotional information to the cortex. A year later, Danish physician Carl Lange (1834–1900) argued – without knowing of James’ ideas – that the tightening and relaxing of the blood vessels (vasomotor system) was the main bodily source of the experience of emotions (Lange, 1885).

British physiologist Charles Sherrington (1857–1952) began to experimentally test James’ and Lange’s ideas towards the turn of the century (Sherrington, 1900: 393). He cut all nerves involved in the sympathetic nervous system in five dogs in his physiology laboratory in Liverpool. The dogs’ brains would then be unable to receive important emotional information from the body. Yet he did not see any change in emotional behaviour in any of the five dogs. Fear, disgust, joy, anger – Sherrington managed to elicit them all with unlessened strength. After this, he also cut the vagus nerve in the neck of two dogs to make them even more ‘insensitive’ to physical changes (Sherrington, 1900: 396). Again, he saw no difference in emotional behaviour (Sherrington, 1900: 402). According to Sherrington, his observations showed that James’ and Lange’s theory was incorrect. However, emotions were not central to his research and he discontinued this line of study.

A few years later, Sherrington stumbled upon emotional behaviour again. He placed this serendipitous finding within the then dominant vertical evolutionary model of the brain. Together with American psychologist Robert Woodworth (1869–1962), he investigated pain pathways in the brain. To do this, they removed the entire cortex in cats. After the operation the cats showed aggressive behaviour. They bared their front teeth, growled, hissed and clacked their jaws. Woodworth and Sherrington called these behaviours ‘pseudaffective reflexes’ (Woodworth and Sherrington, 1904). Keeping in mind Hughlings Jackson’s evolutionarily stratified brain, Woodworth and Sherrington saw their pseudo-affective reflexes emerge when freed from higher, controlling cortical mechanisms (Woodworth and Sherrington, 1904: 234). Woodworth and Sherrington attributed to the cortex an important role in the experience of emotion. Without a cortex, the experimental animal clearly showed an emotional behavioural reflex that was difficult to distinguish from natural emotional behaviour. Yet, according to Sherrington and Woodworth, this occurred without a subjective emotional experience: ‘When the expression occurs it may be assumed that had the brain been present the feeling would have occurred’ (Woodworth and Sherrington, 1904: 234).

With evolutionary theory in mind, a vertical brain theory emerged in the last quarter of the 19th century that localised emotions, low-level drives, primitive behaviour and simple sensorimotor information deep within evolutionarily old(er) regions. As the evolutionarily most recent brain region, the cortex (the more apt term ‘neocortex’ was introduced only in the early 20th century) had a triple function: summative integration of simple(r) sensorimotor information, conscious perception of the urges and emotions, and control over these ‘lower’ urges and emotions. With damage to cortical areas, lower areas operated in unchecked autonomy, as seen in inebriety and neurological and psychiatric disorders. Accidental behavioural findings after decortication of cats by Sherrington (pseudo-affective reflexes) at the turn of the century provided an animal experimental alignment with the theoretical and clinical practice of this vertical evolutionary hierarchical perspective. From 1925, these ideas would begin to be systematically explored by a new generation of physicians and researchers and related to psychoanalytic concepts.

## Emotions (deep) in the brain

Breathing new life into 19th-century research on the relationship between the (autonomic) nervous system and emotional behaviour, American physiologist Walter Cannon (1871–1945) spearheaded a new era of physiological research on emotions from 1925 onwards. He and his collaborators investigated various aspects of sympathetic activity and emotional behaviour by removing the cerebral cortex of cats, as Sherrington had done (Cannon, 1927, 1931; Cannon and Britton, 1925). After removal, they also observed aggressive behaviour, which they called ‘sham rage’. They then placed transections at different subcortical levels to study the effect on behaviour and found that the hypothalamus in particular played an important role in emotions and emotional behaviour (Bard, 1928). Cannon assigned three functions to the hypothalamus: it was the highest motor centre for emotional behaviour and sympathetic activity, it initiated and facilitated impulses to the cortex for emotional experience, and it was a continuous reservoir of emotional and instinctive energy. The cortex controlled these impulses and provided the conscious experience of emotion.

This hierarchical model, given the important role it attributed to the hypothalamus in activity of the autonomic nervous system and emotional behaviour, would have a marked impact on both animal experimental emotion research and explanatory models for treatments such as ECT and psychosurgery in the following years – especially when in the late 1930s American doctors and researchers also related some of Freud’s new ideas to this model. Freud had published a small book entitled *Das Ich und das Es* in 1923 in which he introduced the *id* and *superego* in addition to the *ego* (Freud, 1923). These three psychic functions had a vertical-hierarchical relationship and were in continuous competition with each other (Fancher, 1996: 392–399). As the evolutionarily oldest psychic function, the id was the unconscious source of powerful impulses, such as the need for warmth, nourishment and sexual satisfaction, the libido. Satisfaction of these instincts, however, could come into conflict with demands of the outside world. Therefore, to function socially, these impulses had to be delayed, modified or even suppressed altogether. The superego was that part of the psyche that – fuelled, among other things, by external reality – made moral demands. The ego had the complicated task of integrating the interests of the id, the superego and the social environment into behaviour that allowed instinctive gratification to some extent without a person behaving in a socially or morally reprehensible manner. The id, ego and superego were projected onto two brain regions. The hypothalamus was the source of mostly unconscious emotions, urges and irrational drives – in short, the id; the cortex was responsible for the conscious, rational ego and superego.

This dual concept was used by the American neurologist and psychoanalyst Roy Grinker (1900–1990) in a multi-year study of the effects of shock treatment (psychopharmacological, using metrazol, and/or electrical, using ECT) on mood disorders (Levy and Grinker, 1943; Levy et al., 1941). In the late 1930s, together with the psychoanalyst Helen McLean (1894–1983), he attempted to explain the effects of shock (Grinker and McLean, 1940: 134–138). The core of their theory was the existence of tension between emotional biological drives in the diencephalon (thalamus and hypothalamus) and the suppressive influence of the superego, which would be located in the cortex. Blocked brain pathways were believed to be the cause of psychological symptoms. The ‘exploding’ of connections between the diencephalon and the cortex as a result of the shock would allow pent-up urges and other negative feelings to find a psychological outlet through dreams, thoughts and behaviour. Repeated shock treatment could produce long-term positive effects by permanently changing the pathways between the cortex and the diencephalon in a positive way.

Yet not everyone went along with the idea of the hypothalamus as the main or even only emotion centre in the brain. In the late 1930s, the psychiatrist and psychoanalyst Jules Masserman (1905–1994) aimed to find out whether electrical stimulation of the hypothalamus led to the subjective experience of emotions. To determine this, Masserman first established a number of criteria that he believed were characteristic of true emotional states, such as sensitivity to changes in the environment, a slow extinction of an emotional state, and mutual influences of different emotions. Only when the emotional behaviour of the experimental animals met these criteria, he said, could one speak of real emotions and not pseudo-affective reflexes.

Emotional responses in his experimental cats during electrical stimulation of the hypothalamus did not change when Masserman manipulated environmental influences (Masserman, 1941: 5). They ceased immediately after stopping the stimulation. When he stimulated the hypothalamus during normal spontaneous behaviour, such as drinking milk or licking their fur, hardly anything changed in it. During stimulation, typical emotional behaviours also broke through the normal behaviour, but without stopping the normal behaviour altogether. Finally, after removal of the hypothalamus, he still saw emotional behaviour. According to Masserman, the reactions during stimulation of the hypothalamus were therefore significantly different from real emotions. They were mechanical, stereotypical responses that seemed to have no greater emotional value than the contraction of a skeletal muscle after electrical stimulation (Masserman, 1941: 6). He also stressed that the cortex needs more attention in emotion research (Masserman, 1943: 45). Masserman himself and two colleagues had found that in some animals, simultaneous stimulation of the hypothalamus and part of the somatosensory cortex produced a more intense pseudo-affective response (Masserman et al., 1940: 451). Thus, the cortex here had a facilitating rather than the so often assumed inhibitory role with regard to emotions. The important role of cortical areas on emotional behaviour was also confirmed by Masserman’s finding that the cats in which he had destroyed the hypothalamus still displayed emotional behaviour.

Masserman had shown with his stimulation experiments that the relationship between the hypothalamus, cortex, autonomic activity and emotional behaviour was more complex than stated in Cannon’s model. He thought it was reasonable to conclude that the hypothalamus played a role only in the physical expression of emotions, but it was certainly not the only brain region involved in emotions and emotional behaviour. Others took over from Masserman after the Second World War and developed more extensive models of emotions in the brain.

## The organ language of the visceral brain

Paul MacLean (1913–2007) began his career in Boston in 1946 as a neuroanatomist in the neurophysiology laboratory of neuropathologist and neurologist Stanley Cobb (1887–1967) and the brain wave laboratory of neurologist and electrophysiologist Robert Schwab (1903–1972). MacLean was fascinated by the peculiar combinations of emotional, sensory and physical sensations he observed and recorded in some of his epileptic patients. This inspired him to develop a comprehensive theory of emotion. He had in mind an extension of the ‘mechanism of emotion’ proposed in 1937 by the American neuroanatomist James Papez (1883–1958) of Cornell University (Papez, 1937).

According to MacLean, Masserman’s research had clearly shown that the hypothalamus was an important area for the expression of emotion, and that only the cortex was capable of fully conscious emotional experiences (MacLean, 1949: 338). In evolutionarily ‘higher’ organisms, the cortex increasingly gained influence over behaviour, according to MacLean. Instead of ‘the’ cortex, his theory focused on specific parts of it, notably the gyrus cinguli and the orbitofrontal cortex. These brain regions have many connections with ‘lower’ brain regions that regulate the autonomic nervous system and the viscera, and had also recently been found to be very active in emotions and emotional behaviour. Besides the hypothalamus, other subcortical areas, including certain nuclei in the thalamus, were also given important roles.

MacLean also paid much attention to the evolutionarily older part of the temporal lobe, particularly the hippocampus and amygdala. Several findings had led him in this direction. In his own patients who showed emotional and psychological disturbances (nervous behaviour, obsessive and/or depressive thoughts) between epileptic seizures, he often found a focal epileptogenic zone in the temporal lobe. An experiment conducted by psychologist Heinrich Klüver (1897–1979) and neurosurgeon Paul Bucy (1904–1992) contributed an additional argument. Klüver and Bucy had described the effect of bilateral removal of the temporal lobe in rhesus monkeys in 1937 (Klüver and Bucy, 1937). The animals did not show any fearful or aggressive behaviour afterwards. They approached other humans or dangerous animals without hesitation. They also tended to put anything they came across in their mouths. Whether they had food, dirt, or faeces in front of them, they put it in their mouths without hesitation. Finally, they exhibited awkward sexual behaviour. They masturbated a lot, climbed females and males indiscriminately, and showed bizarre oral-sexual behaviour (MacLean, 1949: 341). To MacLean, this called for research into the relationship between the development of the temporal lobe and the Freudian psychosexual stages of development. Finally, a 1940 experiment by neurologist Ernst Spiegel (1895–1985) and colleagues had shown that bilateral lesions of the amygdala in dogs and cats led to outbursts of anger (Spiegel et al., 1940).

MacLean named his network of brain regions the visceral brain in 1949, because they were intimately linked to various somatic processes and especially to the viscera (MacLean, 1949). In 1952, he renamed his visceral brain the limbic system, because he believed that this network formed a ‘limbus’ (the Latin word for border or edge) between the more primitive and the more advanced parts of the brain (MacLean, 1952).

According to MacLean, the visceral brain played an important role not only in emotions, but also in psychological and psychosomatic problems. For instance, the visceral brain was too primitive to be able to analyse linguistic information, but could well associate symbolically. It could not name the colour red as red, but could relate it to blood, fainting, or aggression. This ability to symbolically connect unrelated phenomena could very well lead to irrational phobias and obsessive-compulsive behaviour. MacLean had also noticed that many psychosomatic patients were extraordinarily intelligent yet emotionally stuck around the oral phase – one of Freud’s psychosexual stages. According to MacLean, the cortex in this group of patients did not have access to visceral brain activity. The emotions that therefore built up unconsciously then found a direct outlet via ‘lower’ visceral brain regions in the form of psychosomatic symptoms, such as peptic ulcer, high blood pressure, or asthma. The stuck emotions had found an outlet through what MacLean called ‘organ language’. For instance, he cited the observation that patients with ulcerative colitis (an inflammatory bowel disease) often showed a tendency towards primitive behaviour (Lindemann, 1945). This could be the result of an unhappy identification with a parental figure full of anger and resentment, leading to what he called ‘visceral turmoil’ and primitive behaviour (MacLean, 1949: 350). Defecation, for example, then became a symbol of expelling the parental figure from the body.

The fundamental emotion research of Woodworth, Sherrington and Cannon would attain clinical relevance from the 1930s onwards. Combining Cannon’s dichotomy of the unconscious and emotional hypothalamus and conscious and rational cortex with psychoanalytic concepts, clinicians sought to explain the effects of biological treatments, such as ECT. From the 1940s, more critical attention began to be paid to this perhaps overly simplistic neural dichotomy of emotion and reason. MacLean, in particular, was influential after the Second World War with his more elaborate and complex scientific and clinical synthesis of the prevailing neurophysiological, evolutionary neuroanatomical, psychosomatic and psychoanalytic thought. His ideas on the visceral brain and limbic system were an important inspiration for brain researchers and psychoanalysts, who in the 1950s were searching for a Freudian interpretation of early experiments with electrical brain stimulation.

## Proust on the operating table

American neurosurgeon Wilder Penfield (1891–1976) had amassed world fame during the 1930s with his treatment of and research into epilepsy at McGill University in Montreal. There, he had further developed a surgical method of his European teacher Otfried Foerster (1873–1941) into a standard procedure for removing epileptic foci (the ‘Montreal procedure’). By asking patients during the operation what they experienced during electrical stimulation and watching carefully what happened to them motorically, neurologically and psychologically, Penfield was able to better localise the epileptic foci on the one hand and leave more healthy brain tissue in place on the other.

In early 1951, Penfield asked New York-based psychoanalyst Lawrence Kubie (1896–1973) to comment on a preliminary version of his lecture for the annual meeting of the American Neurological Association. In this lecture, Penfield (1952) described the vivid memories induced in some of his epilepsy patients during electrical stimulation. Upon stimulation of cortical parts of the temporal lobe, a small number of his patients experienced very vivid moments or events from their past. They moved back into a past situation and experienced the visual, auditory and other sensory impressions as if they were really there again. These experiences stopped immediately when Penfield stopped stimulating, and repeated themselves when he started stimulating again. Penfield noticed that the evoked experiences were ‘a single recollection . . . not a mixture of memories or a generalization’, moreover often trivial in content (Penfield, 1952: 180). They almost always concerned very ordinary aspects of the patient’s daily life, such as a conversation with piano playing in the background, a visit to a bakery, or a popular song of the time.

However, memories were rarely merely trivial from a psychoanalytic perspective, and on reading Penfield’s draft, Kubie became excited about the role electricity could play in the psychoanalyst’s work. In his view, it opened new technological doors for exploring Freud’s ideas about repressed memories in the brain. And that went to the heart of psychoanalysis, because, according to Kubie, almost everything they knew about the causes, treatment and prevention of the problems of their patients was closely linked to the fate of repressed memories. To demonstrate his enthusiasm, Kubie referred to Sigmund Freud and Harvey Cushing (1869–1939), one of the foremost American leaders and experts in the nascent field of neurosurgery in the early decades of the 20th century: ‘I can sense the shades of Harvey Cushing and Sigmund Freud shaking hands over this long-deferred meeting between psychoanalysis and modern neurology and neurosurgery’ (MacLean, 1969: 40).

To learn more about electrical brain stimulation and memories, Kubie travelled to Montreal in the summer of 1951 to observe first-hand Penfield at work (MacLean, 1969: 40–41). But he did more than just observe. Kubie started recording the conversations between Penfield and his patients. To do this, he crawled under the operating table with pen, paper and recording equipment to document literally every spoken word. Soon Penfield also allowed Kubie to conduct psychoanalytic research during operations. While Penfield stimulated electrically, Kubie stimulated the patient to associate freely. Besides the very vivid experiences already described, Kubie distinguished two other forms of electrically induced memories. Some patients experienced events from their past very vividly, but without feeling that they had ‘stepped in’ again. These events remained personal but distant memories. Others experienced not so much specific memories, but more of an externally experienced, generalised abstraction of many specific memories.

In December 1951, Kubie delivered a paper at the Midwinter Meeting of the American Psychoanalytic Association in New York on the implications of brain research for psychoanalysis. He did not shy away from boasting about localisation. The temporal lobe was the place where conscious and unconscious memories converged, like a ‘library of many volumes’ (Kubie, 1953: 26). Kubie referred to MacLean’s theory to explain the vividness of such re-experiences. According to Kubie, the visceral brain acted as a ‘psychosomatic organ’ in unconsciously integrating automatic bodily functions, emotions and (verbal and non-verbal) memories. Of great importance was the idea that memories depended on internal (‘I’) and external (‘non-I’) experiences. MacLean gave near-perfect neurophysiological support to Kubie’s ideas by defining the evolutionarily older cortical parts of the temporal lobe as the ‘crossroads . . . for both internal and external perceptions arising from the eye, ear, the body wall, the apertures, the genitals and viscera’ (MacLean, 1969: 40–41). For Kubie, here, evolutionary and individual development, the functioning of the brain and psychoanalysis coincided seamlessly:

Here then . . . within the temporal lobe and its connections, is the crossroads where the ‘I’ and the ‘non-I’ pole . . . meet. It is impossible to overestimate the importance of this fact that the temporal lobe complex constitutes the mechanism for integrating the past and the present, the phylogenetically and ontogenetically old and new, and at the same time the external and internal environments of the central nervous system. It is through the temporal lobe and its connections that the ‘gut’ component of memory enters into our psychological processes. (Kubie, 1953: 31).

The role of the body in the vividness of re-experiences could not be overestimated:

when electrically evoked experiences are relived most completely the individual . . . gives full somatic participation to the experience. The subject is present in the flesh as well as in the mind. This is ‘gut memory’. . . It is Proust on the operating table; an electrically stimulated *Recherche du temps perdu*. (Kubie, 1953: 46)

What might all this mean for psychoanalytic practice, Kubie wondered (Kubie, 1953: 47). If it could be proved that the memories – so vividly induced by just a small electric current – were repressed memories, it could totally change psychoanalytic practice. By electrically stimulating the temporal lobe, the therapist would be able to elicit repressed memories in a matter of seconds – a process that, in normal psychoanalytic practice, would take days, months, or even years to bring to the surface of consciousness.

Kubie was the first to conduct psychoanalytic research in the early 1950s on some patients who were electrically stimulated in the brain. During stimulation, he saw re-experiences of specific experiences that he believed were stored in the temporal lobe. However, both friend and foe cautioned against making too simplistic a causal link between stimulation of a specific brain region and the patient’s subsequent experiences. The neurophysiologist Warren McCulloch (1898–1969) accused Kubie of falling blindly into ‘the fallacy of simple localization’ (Heims, 1991: 116). Psychiatrist George Mahl (1917–2006) took over the torch from Kubie, trying to avoid the trap of simplistic localisation.

## Drives versus defences

Following Kubie, Mahl wanted to map the various responses during electrical stimulation, from simple physical reactions to abstract symbolic experiences and social attitudes. Together with neurophysiologist José Delgado (1915–2011) and neurosurgeon Hannibal Hamlin (1904–1982), he examined at least four patients between 1953 and 1955 for this purpose. In two, this yielded no significant results (Higgins et al., 1956: 417–418). A man with electrodes in the frontal lobe awaiting a lobotomy showed no changes at all during stimulation. An 11-year-old boy with psychomotor epilepsy showed hardly any changes during stimulation in the temporal lobe. But two other patients did show interesting responses to stimulation in the temporal lobe.

Another 11-year-old boy with psychomotor epilepsy and ‘a somewhat greater tendency towards female identification’ was to undergo surgery, in which the epileptic foci in the temporal lobe would be removed (Higgins et al., 1956: 409–413). A month before the final surgery, Hamlin and Delgado placed multiple electrodes in and on both temporal lobes. The relatively long period prior to the surgery gave Mahl the opportunity to examine the boy extensively.

Mahl had fellow psychiatrist John Higgins conduct six interviews with the boy. Higgins did not know when the boy would be stimulated. The retrospective analysis consisted of a clinical evaluation of the boy’s most salient experiences, behaviours and statements during the interviews.

A number of responses recurred regularly throughout the different interviews. The main physical changes Mahl observed during stimulation were oral sensations. Like some of Penfield’s patients, the boy also had frequent déjà vu-like experiences. He also often became much friendlier in his expressions during stimulation and regularly spoke of a feeling of being alive.

In the fifth interview, something extraordinary happened. Two minutes after the sixth stimulation, the boy mentioned that he wanted to get married. Higgins then asked him with whom. The boy remained silent for some time. Mahl asked Delgado to stimulate again at the same spot. The boy remained silent for over a minute. Then he stuttered: ‘I was thinking – there’s – I was saying this to you. How to spell “yes” – y-e-s. I mean y-o-s. No! “You” ain’t y-e-o. It’s this. Y-o-u.’ Then the boy lapsed into silence again. Mahl asked Delgado to stimulate the same area again. The boy started talking about Higgins’ hair immediately after the stimulation, but changed the subject to pubic hair. That in turn prompted him to talk about sexual games that had been played with him in the past. He then expressed doubts about his sexual identity: ‘I was thinkin’ if I was a boy or a girl – which one I’d like to be.’ Delgado stimulated again. The boy then said seemingly without fear or inhibition: ‘I’d like to be a girl.’

For Mahl and Higgins, this boy’s alternation between male and female ideals was significant. The boy had also expressed male desires during several interviews. For instance, he once compared himself to Superman and at one point wanted to marry an attractive nurse. According to Mahl and Higgins, this male wishful thinking was a defence mechanism to mask his real desire to be a girl. They came to this conclusion also because the boy had already implicitly expressed his desire to be a girl (‘covert feminine strivings’) before the implantation of the electrodes. In the interviews, he had said nothing more about this until the sixth stimulation in the fifth interview.

Did this stimulation produce – as Kubie put it – a direct and vivid re-experiencing of what was stored in the stimulated part of the brain as repressed memories and desires? According to Mahl and Higgins, it was more complex (Higgins et al., 1956: 418). Electrical stimulation in the temporal lobe caused a change in the balance between ‘drive and defence’ in this boy. To confirm this hypothesis, in the sixth and final interview Higgins insisted that the boy express his wish to be a girl without stimulation. This caused embarrassment and reticence in contrast to the candid way he had spoken after stimulation in the fifth interview. Whether stimulation reduced his defence mechanisms or reinforced his deep desire to be a girl – or both – could not be determined from these observations, according to Mahl and Higgins.

This was followed by another extensive study of a 27-year-old housewife with psychomotor epilepsy (Mahl et al., 1964). The woman appeared normal to Mahl and psychiatrist-in-training Albert Rothenberg (1930–). She was of average intelligence, warm in manner and not afflicted with any psychiatric disorder. In the more than two weeks between placement of the electrodes in the temporal lobe and the operation, Mahl was again able to conduct extensive research into the effects of stimulation.

During the stimulation, the woman experienced people appearing in her mind and talking to her. Sometimes this felt just like a thought, sometimes it seemed like a vivid memory, sometimes it was an almost hallucinatory experience. Words also regularly appeared in her mind during stimulation while no one spoke to her. These words were sometimes statements recalled by her, sometimes nonsense words, sometimes swear words, and regularly they were words the woman could not or would not describe. These ego-alien experiences created feelings of fear, diffidence and reluctance to talk about them.

According to Mahl and Rothenberg, one should not see the re-experiencing of memories in this woman as an exact re-experiencing of repressed memories or wishes, just as they had already concluded with the 11-year-old boy. Drawing partly on Freud’s ideas about dreams, they arrived at an alternative hypothesis (Mahl et al., 1964: 360–361). Electrical stimulation of the temporal lobe caused a different state of consciousness, creating a shift in thinking and acting from (conscious and rational) secondary to (unconscious, primitive, magical and associative) primary processes. This had a number of consequences. The conscious and unconscious mental content at the moment of stimulation could be expressed in hallucinatory experiences. Disturbances in perception of the self or the external world could occur. Changes in defence mechanisms could also occur with more primitive and intense manifestations of both unconscious drives and defence mechanisms. What a person experienced, remembered, felt and thought during temporal lobe stimulation was, according to Mahl and Rothenberg, the sum of several variables, and certainly not a direct, electrically induced repressed memory projected brightly, like a film on the screen of conscious perception. Our consciousness was in a different state during stimulation; as in dreams, it was less influenced by psychological defence mechanisms and therefore more receptive to primary processes. The hallucinations, distorted perceptions, irrational content and primitive reactions were therefore a mixture of different types of memory and other mental processes rather than exact trivial re-experiences. This could also better explain the wide variety of experiences that occurred during stimulation.

Mahl and Rothenberg stressed that this type of stimulation experiment should be interpreted with caution (Mahl et al., 1964: 363). Since it involved the dynamic interplay between unconscious drives and defence mechanisms, they saw an important role for psychoanalysts, who knew the pitfalls of interpreting patients’ irrational and primitive utterances. And it was psychoanalysts who could both avoid the pitfall of overly simple and unambiguous conclusions and use their imagination to develop new and interesting interpretations of this complex material.

## Conclusion: From Victorian brains to neuropsychoanalysis

Electrical brain stimulation during neurosurgical operations in the early 1950s offered some psychoanalysts an attractive opportunity for empirical brain research into unconscious psychological processes such as repressed memories, defence mechanisms and repressed (sexual) identity. Their stimulation experiments were influenced by technological, theoretical and therapeutic developments of the 19th and 20th centuries from various disciplines such as evolution theory, brain research, neurology, psychiatry and psychoanalysis. This remarkable, short-lived collaboration – Cushing and Freud shaking hands, so to speak – is thus a compelling example of the pragmatic and eclectic nature that has always characterised a significant part of psychiatric science and clinical practice.

Drawing on evolutionary theory, a vertical theory of the brain emerged in the last quarter of the 19th century that localised unconscious emotions, low-level drives and primitive behaviour deep within evolutionarily older regions. The cortex, as the evolutionarily most recent brain region, provided the conscious experience of and control over these ‘lower’ drives and emotions. In the 1930s, doctors and researchers combined this Victorian brain model with the Freudian tripartite division id, ego and superego. The id was believed to reside in the hypothalamus while the cortex was responsible for the (super)ego. From the late 1940s, neuroanatomist Paul MacLean was to have a great influence on research and clinical practice with his more complex scientific and clinical synthesis of the prevailing neurophysiological, evolutionary neuroanatomical, psychosomatic and psychoanalytic thought. With his theory of the visceral brain, later called the limbic system, he drew attention to the role of certain cortical areas (gyrus cinguli and orbitofrontal cortex) and the temporal lobe (in particular the amygdala and hippocampus) in (unconscious) emotions and emotional behaviour.

Neurosurgeon Wilder Penfield observed unusual experiences in some of his patients during brain surgery. Electrical stimulation of the temporal lobe could lead to simple physical reactions, but also to very specific memories or abstract symbolic experiences. According to some psychoanalysts, this literally opened access to hard-to-reach unconscious psychoanalytic processes. Lawrence Kubie hoped to use electrical stimulation to bring repressed memories back to consciousness. Inspired by MacLean’s theory of the visceral brain, Kubie believed that electrical stimulation of the temporal lobe held the promise of bringing repressed memories back into consciousness and thus drastically shortening the duration of psychoanalysis. George Mahl wanted to use electrical brain stimulation to break down psychological resistance and reduce defence mechanisms. The two psychoanalysts held a different (implicit) view of how the brain and mind worked, however. Kubie assumed a direct causal relationship between the stimulation of a specific brain region and a particular memory. Mahl worked from the idea that electrical stimulation of a specific brain region caused more global changes in consciousness, allowing unconscious irrational and conflictual processes, including memories, to manifest more in consciousness. These experiments in brain stimulation took place in relative academic and clinical isolation. Only a handful of patients were studied, with little impact on either discipline.

The entrenched metaphors that are often used to describe historical developments deserve greater scrutiny. Careful attention must be paid to aspects of history that they do not capture well, as Sadowsky warns us in the discussion of his article ‘Beyond the metaphor of the pendulum: Electroconvulsive therapy, psychoanalysis, and the styles of American psychiatry’ (Sadowsky, 2006: 24–25). He gives a few reasons why we should be wary of the uncritical use of the pendulum metaphor to understand the history of psychiatry. The handful of brain stimulation experiments and their context described in this article provide fertile ground for further research based on Sadowsky’s warning.

First, according to Sadowksy, and as already briefly discussed in the introduction, the metaphor obscures the pragmatic and eclectic nature of psychiatric science and clinical practice. Although there have always been dogmatic reductionists on both sides, many – perhaps even most – psychiatrists have mixed styles in their quest to relieve their patients’ suffering. The studies mentioned in the introduction, which have shown that an important part of psychiatric practice has always been more diverse, eclectic and pragmatic, have described cases of the use of psychoanalytic insights in the application of (ablative) psychosurgery, electroconvulsive therapy, personality disorders and psychopharmaceuticals. The present study on brain stimulation and psychoanalysis adds to the validity and credibility of this perspective, in a triangulation process. It has shown how insights and methods from neurophysiology and neurosurgery have been fluidly mixed with evolutionary, psychosomatic and psychoanalytic theory to gain a better understanding of pathological mental processes.

It would be interesting to support these case studies with quantitative analyses. Digital analyses of, for example, the content of leading journals or medical curricula could provide more general and quantitative insights into the extent of eclecticism and holism in psychiatric science and clinical practice.

Secondly, the metaphor of the swinging pendulum can conceal the continuity of theories and practices, although they appear in other periods in different (sometimes less recognisable) forms. The study of unconscious mental processes is an interesting case in this respect. A connection between unconscious processes and areas deep in the brain was made as early as the last quarter of the 19th century, as this article shows. The first period in which doctors and researchers combined biological and psychoanalytic concepts of the unconscious to understand biological treatment lasted around 15 years, from about the late 1930s to the mid-1950s.

At the same time that external criticism of the scientific and clinical merits of psychoanalysis was growing, a new multidisciplinary discipline, cognitive science, was emerging. Although psychoanalysis became quite unpopular in the emerging cognitive sciences and related neural disciplines, unconscious processes were still being studied in these fields, albeit under a different name. Psychologists had already begun to study the unconscious in their laboratories in the 1930s and 1940s, in an attempt to bring order to the somewhat chaotic clinical realm of unconscious, subconscious, preconscious, foreconscious, superconscious and coconscious phenomena that had developed within the clinical mental sciences since the end of the 19th century (Miller, 1942: v, 1–2). This eventually became a flourishing experimental field of cognitive science, concerned with so-called subliminal processing, or the processing of information below the conscious threshold (Latin: limen) (McConnell et al., 1958; Tsikandilakis et al., 2019).

At the end of the 1990s, the acclaimed neuroscientist Eric Kandel (1929–), as a representative of highly reductionist brain research, inspired by advances in the cognitive and brain sciences, made a new attempt to explicitly revive the cross-fertilisation between psychoanalysis, psychiatry and brain research. His fascination with the biological underpinnings of psychoanalytic concepts dates back to the early 1950s when, as a young medical student, he met Kubie in person. After that, Kandel wanted nothing more than ‘to learn something about where in the brain the ego, the id and the superego might be located’ (Kandel, 2007: 53–55). One of his supervisors advised him to start modestly, with one cell at a time, and then see if he could ever get there. But Kandel recognised his hubris, and over the following decades focused very successfully on mapping cellular changes in the brain during various memory processes, research for which he received the Nobel Prize in 2000.

From the late 1990s onwards, Kandel published a series of papers in which he argued that the cognitive and brain sciences had developed theoretically and methodologically to such an extent that he saw many possibilities for the empirical application of brain research to psychoanalytic concepts (Kandel, 1998, 1999, 2008). He even spoke of ‘biology in the service of psychoanalysis’ (Kandel, 1999: 508). He saw common ground between these two disciplines in unconscious mental processes, in the way mental processes become (causally) connected (‘psychic determinacy’), in fear conditioning and post-traumatic stress, in early life experiences and psychopathological predisposition, and in the biological development of sexual orientation.

Not much later, the neuroscientist Jaak Panksepp (1943–2017) and the neuropsychologist and psychoanalyst Mark Solms (1961–) became important standard bearers of what they called neuropsychoanalysis (Solms and Panksepp, 2012). Panksepp and Solms advocated that more attention be paid to unconscious, affective and instinctive processes within neuroscience – processes that received less attention in strongly cognition-focused brain research. First-person, subjective experiences also had to regain a place in the theorisation and research of brain researchers.

The historiography of psychiatry could benefit from open-minded (interdisciplinary) research into similarities between concepts, perspectives and technologies used within and outside psychiatry. Such a history of psychiatry might then become a less polarised disciplinary history, and probably a more complex one, interwoven with many other scientific and clinical disciplines.

Finally, according to Sadowsky, the metaphor of the pendulum can encourage superficial historical analyses. Simply assuming that the history of psychiatry is one of back and forth does not help us understand the complex interplay of scientific, medical and social factors that influence the changing direction(s) of psychiatry over time. How do technological innovations control this? How do these technological developments interact with social developments? How does psychiatry relate to other scientific and medical disciplines? As an example, Sadowksy notes that the revival of electroshock therapy cannot be explained simply by the revival of biological psychiatry. Why, he asks, have other biological treatments that had disappeared, such as psychosurgery, not been revived? This question underscores the risks of superficiality, because psychosurgery has experienced the same revival as electroshock therapy, although in the form of deep brain stimulation (van der Linden, 2023). This is all the more fascinating because in the 1970s deep brain stimulation was seen as the ultimate lobotomy, a technology that could be used for sophisticated mind and behaviour control, even at a distance using telemetric technology (Breggin, 1983: 372–380). Even more than electroshock therapy and ablative psychosurgery, deep brain stimulation was seen by some critics as the most fundamental technological threat to freedom and autonomy (Breggin, 1983: 380).

This raises the question of how and why deep brain stimulation re-emerged as a therapy of last resort some 20 years ago. The answer lies in a complex of interacting variables, ranging from societal, technological and medical developments to the perspective of the individual patient. Relevant developments include the dominance of biological perspectives within psychiatry, the societal fascination with the brain catalysed by new brain imaging techniques, and advances in the treatment of neurological disorders such as Parkinson’s disease (Benabid, 2003). Its resurgence also seems to be linked to some apparent advantages over electroshock therapy. Deep brain stimulation held the technological (and commercial) promise of providing highly localised, seizure-free electrical manipulation of the brain, replacing jolts of electricity coursing through the entire brain and resulting in a complete shock. There are patients who prefer the invasive neurosurgical procedure of deep brain stimulation to electroconvulsive therapy for these reasons (Filkowski et al., 2016: 126). Thus, the promise of technological precision aligned doctors, researchers, companies (such as Medtronic, St Jude Medical, Neuropace and recently Neuralink) and patients.

Moving away from the pendulum metaphor opens up new ways of looking at how psychiatric patients have been understood and treated over time. It reveals psychiatry as a diverse, holistic and eclectic medical discipline with ever-changing relationships with other scientific, medical and clinical disciplines and with society. Such a dynamic, complex and contextual historical approach – we could use the network as a metaphor for this perspective – frees the historian to search for new and unexpected collaborations, theories and methods of understanding and treating psychiatric patients, as this article has aspired to exemplify.

Finally, it should be noted that while the simple adoption of the pendulum metaphor risks falling victim to superficial historiographic narratives, moving away from it does not automatically lead to good scholarship. Each metaphor highlights certain aspects of the past and obscures others, and can be used for in-depth or superficial research.

There is still no generally accepted biological mechanism that explains psychiatric disorders. Moreover, psychiatry and psychology lack generally accepted explanations for the effectiveness of biological treatments, such as antidepressants, electroconvulsive therapy and electrical (deep) brain stimulation. The special collaborations between psychoanalysts, neuroscientists and doctors described in this article have not (yet) changed this. That said, the open and holistic approach to the study and treatment of psychiatric disorders, as exemplified by these scientists and clinicians, is a laudable attitude, as it does justice to the complex stratification of psychological processes. If historians of psychiatry also work from the same perspective, medicine and history can thus provide a force against the pendulum swinging back towards oversimplified models of our minds, behaviour and history.
